# Effect of Hydration Time in Saline on the Swelling and Uniaxial Tensile Response of Annulus Fibrosus of the Intervertebral Disc

**DOI:** 10.3390/jfb16100365

**Published:** 2025-10-01

**Authors:** Małgorzata Żak, Sylwia Szotek

**Affiliations:** Department of Mechanics, Materials and Biomedical Engineering, Faculty of Mechanical Engineering, Wrocław University of Science and Technology, 50-371 Wrocław, Poland; sylwia.szotek@pwr.edu.pl

**Keywords:** intervertebral disc, annulus fibrosus, swelling, hydration, mechanical properties

## Abstract

The intervertebral disc (IVD) is a biphasic tissue in which the extracellular matrix (ECM) acts as a structural scaffold and regulates hydration and solute transport. The influence of hydration on the swelling and mechanical properties of the IVD, particularly the annulus fibrosus (AF), is not fully described in the literature. Hydration is assumed to affect inter- and intramolecular hydrogen bonding and hydrophilic interactions, thereby modulating tissue mechanics. This study aimed to assess the effect of hydration time on free swelling of AF and its impact on mechanical performance. AF specimens were divided into five groups, hydrated for 0, 10, 20, 30, or 40 min and subjected to uniaxial tensile testing until failure. Swelling-related geometric changes were correlated with tensile properties. Results demonstrated that hydration duration significantly influenced AF’s structural and mechanical characteristics in anterior and posterior IVD regions. Hydration increases rapidly within 10–20 min, causing cross-sections to swell, stress capacity to decrease, and stiffness to remain unchanged. However, after 40 min, the tissue becomes swollen beyond physiological balance. These findings identify hydration duration as a critical factor regulating AF function and provide important insights for experimental standardization, numerical modeling, and hydrogels designed for intervertebral disc regeneration.

## 1. Introduction

Elements of the intervertebral disc (IVD), i.e., outer annulus fibrosus (AF) and centrally located nucleus pulposus (NP), are biphasic mixtures in which composite networks of collagen fibers are embedded in an extracellular matrix consisting of water, proteoglycans, and elastin fibers. Collagen proteins form densely arranged fibers, whose structure is dominated by collagen type II and, to a lesser extent, collagen types I and III [1,2,3,4,5]. The network of collagen fibers determines the mechanical tensile and compressive strength of the IVD. The extracellular matrix (ECM), on the other hand, which is a multi-component, ordered, and elastic structure with gel properties, ensures the integrity of the IVD structures and also determines its mechanical properties. The ECM is not only a static scaffold for cellular elements and collagen fibers but also a reservoir of numerous cytokines involved in physiological processes, such as migration, adhesion, differentiation, and mutual interactions [1,6]. We must also note its significant role in maintaining proper hydration and transport of solutes in the IVD [7,8].

Water accounts for 60–90% of the volume of the IVD [9,10], and maintaining its proper levels is possible thanks to the diffusive flow of substances at the endplate (EP) border and, to a lesser extent, the peripheral flow from the surrounding blood vessel. Water accumulation is mostly determined by the proteoglycan, which binds free water molecules in extrafibrillar spaces, while in protein structures, such as collagen fibers, proteoglycans form intrafibrillar bonds with water molecules [11]. In a spine subjected to continuous complex cyclic loading (mainly compression and flexion), the IVD naturally regulates the level of hydration through the so-called swelling pressure [12,13].

In the case of experimental studies, the most common way to restore proper hydration of the IVD is to immerse it in a hydrating solution for a specific period of time [14,15]. However, despite many studies, no single consistent protocol has been developed to obtain physiological hydration in the studied IVDs. The differences usually relate to the following: the hydration substance used (usually saline or phosphate-buffered saline), hydration method (e.g., by wrapping or immersion), and, most importantly, the length of the hydration time. In the case of the complete functional spinal unit (FSU), restoration of the established physiological hydration of the human IVD occurs within 6 h of immersion in normal saline at a temperature of 36 °C [16,17]. The latest reports also indicate that, after one hour of immersion of the FSU in a hydrating solution, the IVD shows excessive swelling [15], which results in a large scatter in the obtained results regarding mechanical properties [13,16,18]. There are also doubts about the hydration conditions in studies on isolated fragments of the AF. Analysis of the effect of hydration on the mechanical and structural properties indicate that the maximum absorption time of a hydrating solution by an AF fragment should not be shorter than 30–45 min [18,19,20,21,22]. The influence of hydration on swelling and mechanical properties of both the entire IVD and isolated AF fragments is not fully understood, and the theoretical assumption is that in tissue materials hydration affects inter- and intra-molecular hydrogen bonds and hydrophilic interactions. The visible volumetric deformation under the influence of a hydrating solution is interpreted as an effect of the stabilization of hydrated hydrogen bonds [12,16].

Earlier research by Żak et al. [18] on the effect of free hydration of the AF showed unequivocally the existence of a preferential swelling direction affecting the value of the cross-sectional area of the specimens and, thus, the obtained mechanical parameters. However, the above study was limited to the analysis of isolated fragments of the AF, mainly in the non-hydrated and fully hydrated states (for a minimum of 30–45 min) without checking whether indirect changes in water content could significantly define the properties of the examined tissue. Therefore, the aim of this study was to assess the effect of hydration time on the free swelling of the AF and to determine whether it has a significant effect on its mechanical properties. The conducted tests included AF specimens surrounded by fragments of bone elements (EP and vertebral body), thus preserving physiological anchoring of the AF to the vertebrae.

There is still a lack of knowledge in the literature regarding the swelling behavior of the annulus fibrosus and how this directly influences its mechanical properties. From a clinical perspective, hydration-dependent changes in AF function are essential, because they may contribute to disc degeneration, altered load distribution, and impaired biomechanical performance of the spine. Therefore, understanding the role of hydration is relevant for basic biomechanics and improving experimental protocols, developing accurate numerical models and hydrogels designed for intervertebral disc regeneration. Based on this background, we hypothesized that hydration time critically modulates the swelling and tensile response of the annulus fibrosus. In particular, we assumed that intermediate hydration states, resulting from different durations of exposure to saline, could significantly affect the mechanical parameters of the AF and, thus, determine its functional performance.

## 2. Material and Methods

Fifty fresh FSUs were collected from the thoracic spine (Th3 ÷ Th12) of six domestic swine [23], obtained from a local slaughterhouse, aged around nine months and weighing on average 100 kg. Each FSU consisted of two halves of the vertebral bodies and the IVD between them, which were stored in separate plastic bags at −20 °C until testing.

### 2.1. Specimen Preparation

Prior to testing, each FSU was thawed at room temperature and cleaned to expose individual parts: the vertebral body and the IVD. Previous research proves that a single slow freezing and thawing cycle does not affect the loss of hydration by the FSU structures and does not change the mechanical properties of the IVD [15,24].

**Figure 1:**
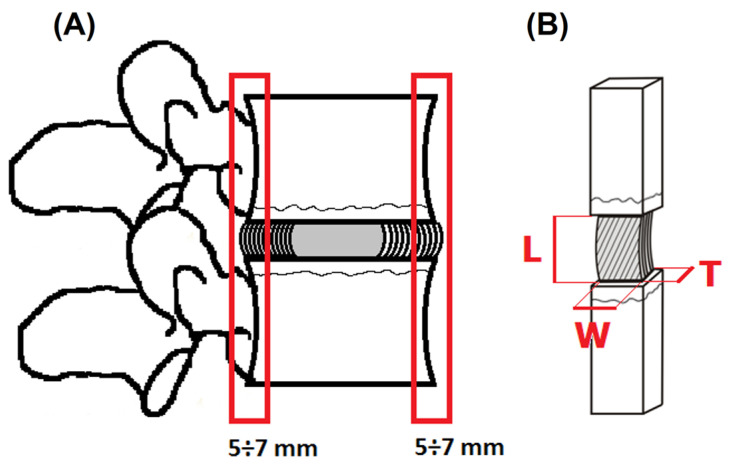
Diagram of the specimen preparation: (**A**) anterior and posterior parts of IVD; (**B**) geometric dimension of multilayer AF specimens with a fragment of the EP and vertebral body: L—length, W—width, T—thickness.

From this preparation, 5–7 mm thick anterior and posterior parts of the IVD were excised (with a fragment of the EP and vertebral body), which were then divided sagittally into two symmetrical specimens (Figure 1A). A total of 100 multilayer AF specimens were prepared (from the anterior part, *n* = 50; from the posterior part, *n* = 50).

Each specimen was prepared in such a way as to obtain similar initial geometric dimensions of the AF from the anterior and posterior parts of the IVD: length, width, and thickness (Figure 1B). Macroscopic analysis was carried out to determine the geometric dimensions of individual samples. Images were recorded using a SteREO Discovery V20 stereomicroscope (made by Zeiss, Jena, Germany). Changes in geometric features were quantified based on measurements along the edges of the tested samples. The final result was the arithmetic mean of five independent measurements taken on the surface of each sample. The initial mean values of the obtained dimensions of the AF are shown in Table 1. The specimens were stored in separate plastic bags at a constant humidity and ambient temperature until testing.

This way of maintaining environmental conditions does not significantly affect the water loss by the specimen, as confirmed by Adams and Green [25], who stated that the daily loss of hydration is half as large again as the loss in dissected post-mortem specimens.

### 2.2. Hydration

The effect of hydration time on swelling and mechanical parameters of the AF of the IVD was studied in a hydration medium of 0.9% normal saline, most commonly used in examination of tissue material. The temperature of the salt solution was maintained at a constant level and, similar to the tested samples, corresponded to the ambient temperature. The literature indicates that the full saturation time of multilayer specimens of the AF of the IVD should not exceed 45 min [18,26]. Therefore, in this study, the specimens were divided into five groups (*n* = 10): a non-hydrated group (*t* = 0 min) and four groups that were hydrated for times (*t*) of 10, 20, 30, and 40 min, respectively. Each sample was placed in an individual, tightly closed vessel containing an equal volume of physiological saline solution.

For each group, water absorption capacity was determined from the change in the weight of the specimens during hydration [18]. Each sample was weighed on a Radwag PS 1000/C/2 precision balance with an accuracy of 0.001 g, both before and during hydration. The percentage of hydration (Δ*H*) determined as the increase in the specimen’s weight was defined according to the formula

(1) $$∆H=\frac{m\left(t_{n}\right)-m(t_{0})\;}{m(t_{0})}\;,n\epsilon \{10,20,30,40\}$$

where Δ*H* [mg/mg] is hydration, *m*(*t*_*n*_) [mg] is weight of the specimens during of hydration time *t_n_* [min], and *m*(*t*_0_) [mg] is starting weight of the specimens *t*_0_ = 0 [min].

In parallel with these tests, the geometric dimensions of the AF (length, width, and thickness) were also measured to determine the effect of hydration on the swelling of multilayer AF specimens embedded in bone attachment. Based on the obtained data, the following areas were determined:

- Cross-sectional area (*A_C_*) as the product of the width and thickness defining the changes in the radial direction of the IVD;
- Longitudinal sectional area (*A_L_*) as the product of the length and width defining the changes in the circumferential direction of the IVD.

### 2.3. Mechanical Test

Individual specimens from each group were analyzed for mechanical properties in a uniaxial tensile test. The uniaxial tensile test was performed using an MTS Synergie 100 testing machine until specimen failure. Bone elements were placed in specially designed grips that prevented the specimen from slipping during the test. Initially, each specimen was subjected to 5 conditioning cycles followed by the actual uniaxial tensile test at a constant rate of 0.5 mm/s. A time interval was observed between the tests of individual specimens so as to maintain the same conditions at each testing stage of all groups. Based on the obtained force–displacement and stress–strain curves, the following values were determined: maximum force (*F_MAX_*), stiffness coefficient (*k*), ultimate tensile strength (*σ_MAX_*), and Young’s modulus (*E*). The values *k* and *E* were determined from the slope of the rectilinear parts of the characteristic curves in the range of 35–70% [18,25,26].

### 2.4. Statistical Analysis

The results were presented as mean values with standard deviations. The statistical analysis was performed using one-way analysis of variance (ANOVA) for independent samples at a statistical significance level of 0.05. Mean values between the groups were compared using Tukey’s multiple comparison test. In addition, a linear correlation was assumed for the obtained geometric values and mechanical parameters depending on the hydration time, determined based on the correlation coefficient R with the value |R| ≥ 0.70 considered as a strong correlation.

## 3. Results

### 3.1. Hydration

**Figure 2:**
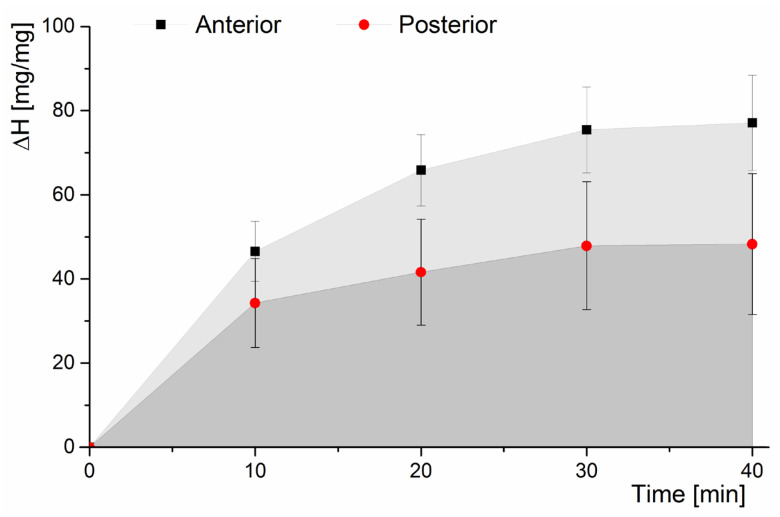
Characteristic hydration changes with time for multilayer AF specimens with a fragment of the EP and vertebral body from anterior (black squares, light grey area) and posterior (red circles, dark grey area) parts of the IVD.

The hydration progress of multilayer AF specimens was the same in the anterior and posterior parts. The change in hydration over time was consistent with the data presented in the literature [18,26] indicating that the rate of water absorption is greatest in the first 30 min and decreases over a longer hydration time (Figure 2).

After the first 10 min, hydration (Δ*H*) increased by 46% in the anterior part and by 34% in the posterior part compared to the initial value. In the subsequent three hydration periods of 10 min each, the increase in hydration was much smaller. After 40 min, hydration of the AF amounted to 77% in the anterior part and 48% in the posterior part. The Δ*H* value was lower in the posterior part than in the anterior part due to differences in the AF structure.

The width and thickness of the AF specimens increased significantly with hydration time (Table 1). The increase in the width of the tested specimens was linear and amounted to a maximum of about 23% both in the anterior and posterior parts. At the same time, statistical analysis showed no differences at a significance level of 0.05 between the width values obtained in the examined hydration times. By contrast, statistically significant differences were found when analyzing the thickness of the specimens. In the anterior part, the initial thickness was 1.04 ± 0.28 mm and increased slightly during the first 20 min of hydration. Statistically significant increases were found after 30 min of hydration, where AF thickness increased more than twofold and after 40 min of hydration where it increased more than threefold.

**Figure 3:**
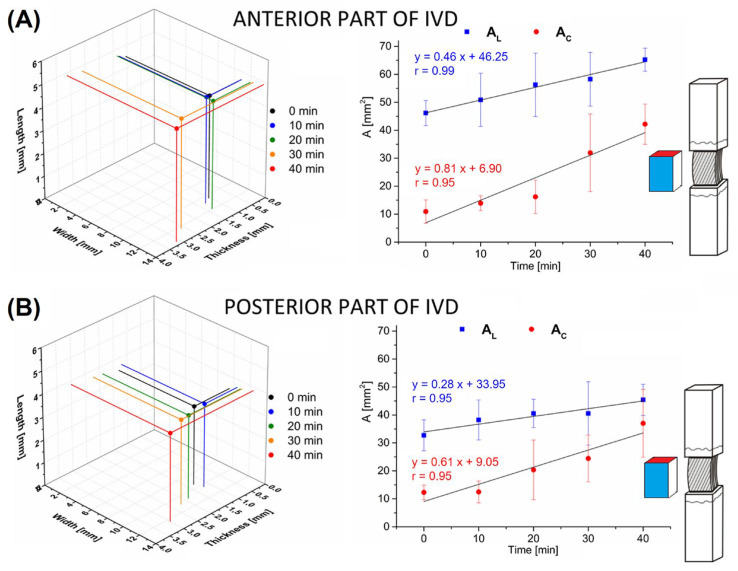
Average values of the cross-sectional area (*A_C_*) and longitudinal sectional area (*A_L_*) resulting from the change of geometric dimensions of multilayer AF specimens depending on the hydration time from: (**A**) anterior part of IVD; (**B**) posterior part of IVD. Linear correlations between *A_C_* and *A_L_* at hydration time.

In the posterior part, the initial thickness was 1.30 ± 0.27 mm, and, as in the anterior part, it increased slightly over 20 min of hydration. After 30 min of hydration, the AF doubled in thickness. A statistically significant increase was also found after 40 min of hydration, where there was an almost threefold increase in AF thickness. The determined values of width and thickness also significantly influenced the values of the cross-sectional area (*A_C_*) and longitudinal sectional area (*A_L_*) of multilayer AF specimens with bone attachment, as shown in Figure 3. In the anterior part, the initial value of *A_C_* was 10.91 ± 4.17 mm^2^ and increased linearly by 74% (R = 0.95) with hydration to the value of 46.16 ± 4.48 mm^2^ for *t* = 40 min. In the case of *A_L_*, the initial value was 42.19 ± 7.18 mm^2^ and increased linearly by 29% (R = 0.99) with hydration to the value of 65.25 ± 4.11 mm^2^. In the case of the AF from the posterior part of the IVD, there was also a linear increase in the values of *A_C_* and *A_L_* with hydration time. The initial value of *A_C_* was 12.31 ± 2.60 mm^2^ and increased by 67% (R = 0.95) for time *t* = 40 min (37.00 ± 12.12 mm^2^). The value of A_L_ for *t* = 0 min was 32.73 ± 5.56 mm^2^ and increased by 28% (R = 0.95) for *t* = 40 min (45.40 ± 5.57 mm^2^).

### 3.2. Mechanical Test

**Figure 4:**
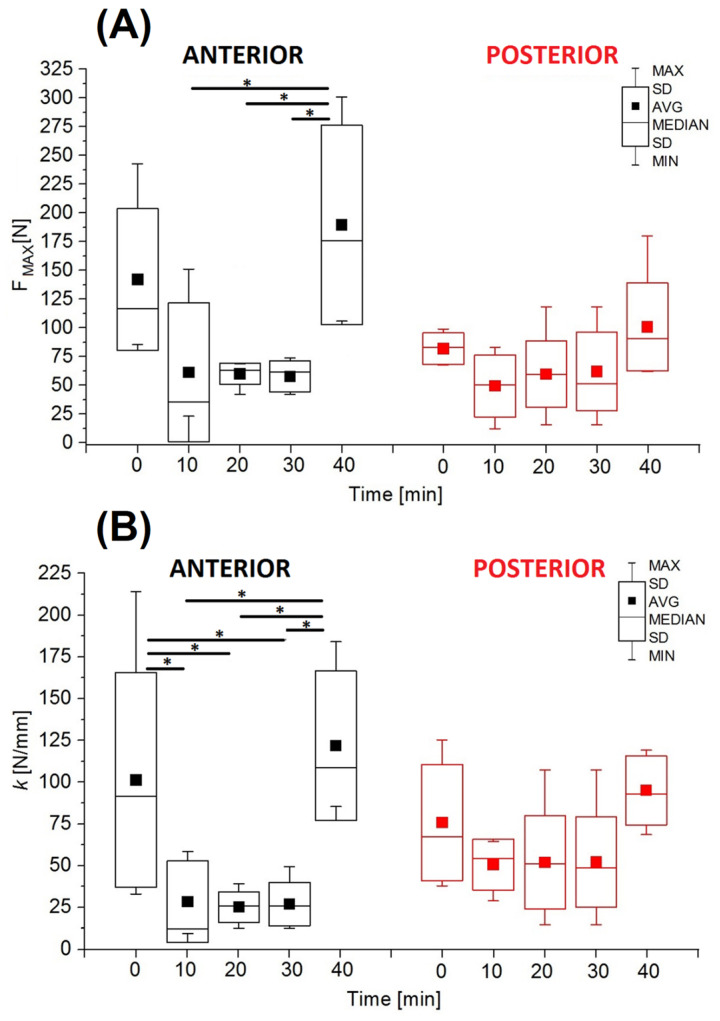
The mechanical parameters of multilayer AF specimens from anterior and posterior parts of IVD during hydration time: (**A**) maximum force (*F_MAX_*); (**B**) stiffness coefficient (*k*) (* *p* < 0.05).

The analysis of the mechanical parameters showed that, in the anterior part, the maximum force (*F_MAX_*) in the non-hydrated group (*t* = 0 min) was 142.10 ± 61.66 N (Figure 4A). By contrast, groups with hydration times between 10 and 30 min showed a marked decrease in the *F_MAX_* values. After 40 min of hydration, the maximum force was 189.54 ± 86.83 N and was 25% higher than in the non-hydrated group and about 70% higher than in the groups with hydration times between 10 and 30 min.

In the posterior part, the maximum force was significantly lower than in anterior part (Figure 4B). At the same time, also in the posterior part, differences in the *F_MAX_* values between the groups were not statistically significant. The *F_MAX_* value in the non-hydrated group (*t* = 0 min) was 81.82 ± 13.72 N and was higher than the values in groups with hydration times between 10 and 30 min, for which the values of this parameter remained similar. For hydration lasting 40 min, the maximum force was 19% higher than in the non-hydrated group.

**Figure 5:**
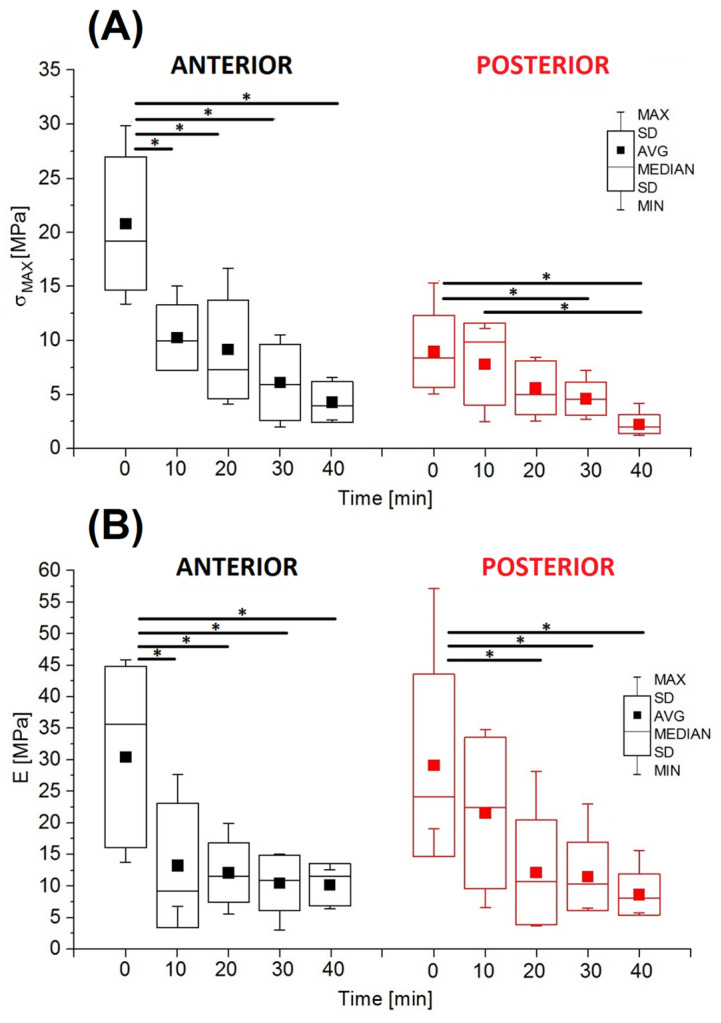
The mechanical parameters of multilayer AF specimens from anterior and posterior parts of IVD during hydration time: (**A**) ultimate tensile strength (σ_MAX_); (**B**) Young’s modulus (E) (* *p* < 0.05).

Statistically significant differences were found between ultimate tensile strength (*σ_MAX_*) and Young’s modulus (*E*). In both studied areas (anterior and posterior), the analyzed parameters were characterized by a decrease in values with increasing hydration time (Figure 5). In the anterior part of the AF, ultimate tensile strength in the non-hydrated group was 20.82 ± 6.17 MPa and was statistically significantly higher than the values obtained in the other groups (Figure 3A). The σ_MAX_ values after 10 and 20 min of hydration (10.26 ± 3.04 MPa and 9.17 ± 4.59 MPa, respectively) were more than half lower than the value in the non-hydrated group (*t* = 0); after 30 min of hydration, ultimate tensile strength was 71% lower (10.26 ± 3.04 MPa), and after 40 min it was 79% lower (9.17 ± 4.59 MPa). In the posterior part of the AF, ultimate tensile strength in the non-hydrated group was 8.98 ± 3.35 MPa and was higher than the values obtained in the other groups.

Similar to the analysis of values of ultimate tensile strength, it was shown that the values of Young’s modulus in the anterior part of the AF were statistically significantly higher in the non-hydrated group (20.82 ± 6.17 MPa) than in the other groups (Figure 5B). The E value after 10 min (10.26 ± 3.04 MPa) was lower by 56%, while for other hydration times it was lower by over 60–67%. In the posterior part of the AF, there was also a visible decrease in the E value with increasing hydration time, with statistically significant differences between the non-hydrated group (29.11 ± 14.47 MPa) and the groups hydrated for 20, 30, and 40 min, respectively. The values of Young’s modulus in the posterior part of the AF also decreased with increasing hydration time. At the same time, the mean *E* values in the individual groups in the posterior part were similar to those in the anterior part of the AF.

## 4. Discussion

The results of this study research describe the effect of hydration on the swelling and mechanical properties of the AF, which consequently determine the behavior of the entire IVD. Analysis of hydration values (Δ*H*) showed that AF specimens absorb water to a much lesser extent in the posterior part than in the anterior part due to the differences in the structure of the IVD. Additionally, it should be noted that leaving a specimen in the hydrating solution for 10 min increased hydration by 46% in the anterior part and by 34% in the posterior part (Figure 2). After 40 min, hydration increased by 77% in the anterior part and by 48% in the posterior part. Such a large increase in hydration does not correlate with data on daily water loss by the loaded IVD. Adams and Hutton showed in their study that compression loading contributes to the loss of 10–15% of the hydration of the human IVD, which is consistent with the results of Kraemer et al. [27] indicating a loss of 8–11% per day [28]. On the other hand, McMillan et al. [29] showed that the FSU subjected to cyclic flexion loading with a force of 1500 N for 6 h induced a loss of hydration in the AF not exceeding 30% in the anterior and posterior parts of the IVD and 20% in the NP area. Therefore, already at this stage of evaluation of the obtained values of the hydration increase, we can conclude that, in the case of isolated multilayer AF specimens, the time to restore the so-called ‘environmental conditions’ should be much shorter than that indicated so far in the literature (not shorter than 30 min).

The evaluation of the effect of hydration time on swelling of the AF also showed that significant geometric changes of the specimen concern mainly its thickness and, to a lesser extent, its width and length (Table 1), and thus the dimensions related to the radial and circumferential directions of swelling of the IVD [18]. The initial value of the AF height (non-hydrated group for *t* = 0 min) in the anterior part (4.51 ± 0.69 mm) was greater than the value in the posterior part (3.46 ± 0.59 mm), which is consistent with differences in the anatomical structure of the IVD (Figure 3). The value of the AF length during hydration increased slightly in both examined areas: anterior (about 7%) and posterior (about 10%). The lack of a marked increase in height may have been due to the presence of bone elements (fragments of the vertebral body), which limited the change in the axial dimension of the AF as a result of the flow of the hydration fluid along the long axis and the highly probable swelling of the cartilage covering the surfaces of the EP. This highly simplified method was used to recreate some physiological behavior of the AF, while minimizing the increase in geometric parameters of the height of the AF. Maintaining the interface between the AF and the osteocartilaginous elements, thus, allows comparison of the obtained results with the conditions of free swelling of the IVD limited in the FSU by two vertebral bodies. The lack of limitation of the multilayer AF structure during hydration results in a significant increase in the height and a decrease in the width of the specimen, as presented in a study by Żak et al. [18]. As a consequence, this leads to excessive and non-physiological swelling causing a rearrangement of collagen fiber bundles and a change in their tensile stress, thus affecting the anisotropic properties of the AF [13,16,20,21,30,31]. In an isolated AF (even with bone attachment), the effect of osmotic pressure exerted by the NP towards the AF is obviously neglected. It is worth noting here that, as reported by Costi et al. [15], already after one hour of hydration of the FSU, hyperphysiologic swelling occurs [31], causing an increase in the osmotic pressure of the IVD and its negative impact on the conducted mechanical tests. Osmotic pressure together with axial loading contributes to the stretching of collagen fiber bundles in the AF and its bulging in the radial direction, and the resulting hyperosmotic loading causes a reduction in the amount of absorbed water and an increase in IVD stiffness. Bezci et al. [13] indicate that strain and material properties of the IVD are largely dependent on osmotic loading, and the increase in stiffness is due to material compaction and its nonlinear mechanical behavior.

**Figure 6:**
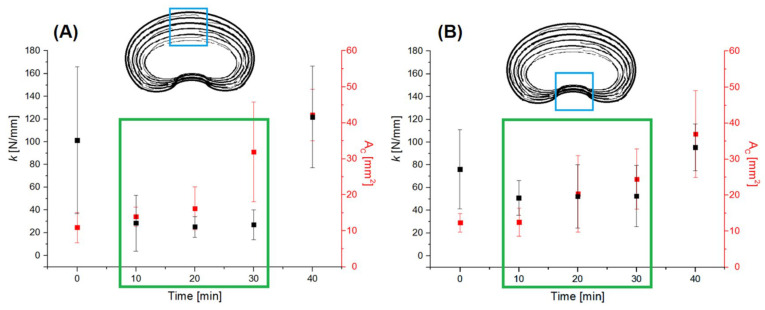
The characteristics of the stiffness coefficient—cross-sectional area (k–A_C_) of multilayer AF specimens depending on the hydration time from: (**A**) anterior part of IVD; (**B**) posterior part of IVD. The green box highlights the range of hydration times (10–30 min) in which stiffness values did not increase significantly despite a linear increase in cross-sectional area. The blue box in the schematic cross-sections indicates the analyzed region of the annulus fibrosus (AF) from which the specimens were collected and tested.

The analysis of the mechanical parameters of the AF (obtained in the presented research) independent of the geometric dimensions, such as maximum force and stiffness coefficient (Figure 4), shows that both non-hydrated specimens (*t* = 0 min) and specimens hydrated for *t* = 40 min were characterized by much higher values than in the case of hydration for *t* = 10–30 min. For the hydration time *t* = 10–30 min, there was no significant increase in stiffness despite a linear increase in the cross-sectional area (A_C_), which was characteristic of the AF both in the anterior and posterior parts of the IVD (Figure 6).

This means that the increase in the stiffness of the IVD did not result from an increase in osmotic pressure and its impact but to a large extent from volumetric changes alone in the structure of the AF caused by hydration. The relationships presented in Figure 6 show that the stiffness of the material depended on the volume of collagen fibers in the matrix, i.e., the extracellular matrix (ECM). The tight spaces between collagen fibers and between individual lamellae are filled with the ECM, which binds together bundles of fibers and lamellae, preventing them from buckling [32].

AF showed low material porosity due to the initial high stiffness (for *t* = 0) at a relatively high fiber density in the test specimen. In addition, despite the shortest possible direct contact with air to minimize its impact, the outermost structures of the AF may have been dehydrated, as also evidenced by the largest increase in hydration in the first 10 min. The absorbed water was stored primarily as free (unbound) water in interfibrillar spaces that contain the ECM [11]. The decrease in stiffness and lack of further significant changes in the range of 10–30 min of hydration were due to the diffusive flow of water into the deeper structures of the AF. During this time period, according to the progress of hydration (Figure 2), the incremental percentage change in hydration (Δ*H*) was much smaller than for the first 10 min. Further hydration led to hyperphysiologic swelling as a result, of which accumulated water in the ECM exerted pressure on the bundles of collagen fibers within it. At this stage, it was the matrix, i.e., the water-saturated ECM, that determined the mechanical parameters of the specimen, which was also indicted by the observed decrease in ultimate tensile strength (Figure 5). Thus, hyperphysiologic swelling affects not only the osmotic pressure of the NP but also determines the stiffness of the AF.

**Figure 7:**
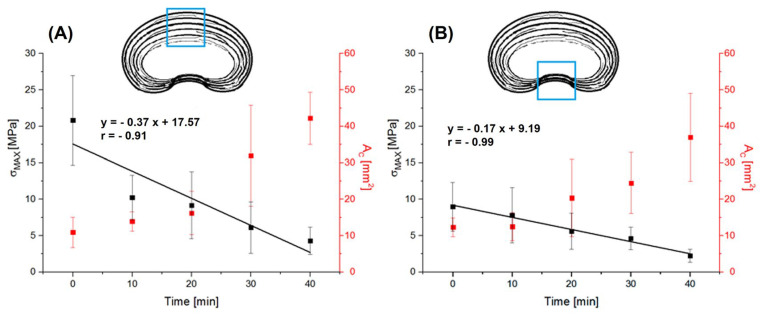
The characteristics of the ultimate tensile strength (σ_MAX_)—cross-sectional area (σ_MAX_—A_C_) of multilayer AF specimens depending on the hydration time from: (**A**) anterior part of IVD; (**B**) posterior part of IVD. The blue box in the schematic cross-sections indicates the analyzed region of the annulus fibrosus (AF) from which the specimens were collected and tested.

As has been shown, the main changes in the cross-sectional area are related to the increase in thickness due to the accumulation of water between the connections of individual lamellae, causing a decrease in collagen fiber density (increase in material porosity) in the increasing volume of the specimen. The test results indicate that this affects the obtained stress value. By relating the linear increase in the cross-sectional area (*A_C_*) to the mechanical parameters dependent on it, we can see that ultimate tensile strength (*σ_MAX_*) decreased linearly in the anterior part (R = −0.91) and in the posterior part (R = −0.99) with hydration (Figure 7). The stress values arising in the outer lamellae of the AF were higher in the anterior part (Figure 7A) compared to the posterior part (Figure 7B).

The AF fibers oriented at ±30° allow the IVD to withstand the generated tensile stress, while the task of the NP and the inner AF lamellae (with predominantly ECM content) is to withstand the compressive force [33]. The increase in water content contributed to a reduction in the density of collagen fibers, thus causing them to stretch as a result of swelling of the AF, which in turn led to an increase in the porosity of the AF and a gradual decrease in its strength.

### Limitations

Although this study provides new information on the effect of hydration time on the mechanics of the annulus fibrosus, several limitations should be noted. Uniaxial tensile testing of AF samples bonded to bone is a simplified load model. It does not fully reflect the complex physiological conditions of the intervertebral disc in vivo, which include cyclic compression, torsion, shearing, and the influence of osmotic pressure generated by the nucleus pulposus. In addition, porcine discs were used as a model, which, although structurally similar to human intervertebral discs, do not fully reflect the heterogeneity and degeneration-related changes found in human tissue.

Despite these limitations, the results are very significant. From a basic research perspective, they provide new evidence of the effect of hydration on AF swelling and its tensile properties, thus filling a gap in the literature and contributing to the standardization of hydration protocols for experimental biomechanics. Beyond basic science, the findings are relevant for improving boundary conditions in computational modeling, guiding preclinical research strategies and supporting the development of hydrogels designed for intervertebral disc regeneration, directly relevant for therapeutic applications to restore physiological disc function.

## 5. Conclusions

In conclusion, the research results presented in this article show a significant effect of hydration time on the mechanical and structural parameters of the AF, which was confirmed both in the anterior and posterior parts of the IVD. The optimal conditions for the AF subjected to hydration occur at a hydration time of no more than 10–20 min. In this time range, there was no significant increase in stiffness despite a decrease in the AF stress. Hydration of an isolated AF specimen with bone attachment by immersion in normal saline indicates that peripheral diffusive flow is of equal importance to that occurring at the EP border. Hyperphysiologic saturation of the IVD with a hydrating solution induces volumetric changes largely due to changes in the cross-sectional area, resulting in increased porosity of the AF, and thus increased stiffness and impaired strength. The impaired condition of the collagen fiber network weakened by hydration affects the degree of anisotropy of the composite AF structure. This also demonstrated that the contribution of the ECM has a significant effect on the mechanical properties of the IVD and process of damage formation leading to its degeneration.

The sensitivity of the collagen fiber bundles forming the individual AF layers to peripheral and radial swelling during hydration makes it difficult to avoid local tensile stress concentrations, where some bundles of adjacent fibers are stretched transversely to the fiber axis. Such loading causes relatively easy rupture of the connection at the boundary between the fibers and the matrix, i.e., the ECM, which is particularly unfavorable and leads to the penetration of the NP towards the area of load-bearing layers, which are the outer layers of the AF.

The presented research contributes significantly to the knowledge about the effect of hydration and the resulting swelling on mechanical properties, as well as helps to understand the damage mechanism in the IVD. Consequently, it will allow to improve the protocol for selecting boundary conditions both in experimental studies and in material models for numerical simulations. At the same time, it provides important information for work on the use of hydrogels in which the dispersed phase is water and which are increasingly used in implants that are becoming increasingly better at imitating the behavior of the physiological IVD.

## Figures and Tables

**Table 1:** The geometric dimensions of multilayer AF specimens from anterior and posterior parts of IVD during hydration time.

Region	Geometric Parameters	Hydration Time [min]				
		0	10	20	30	40
A	L [mm]	4.51 ± 0.69	4.66 ± 0.44	4.71 ± 0.39	4.77 ± 0.26	4.88 ± 0.63
P		3.46 ± 0.59	3.65 ± 0.42	3.68 ± 0.36	3.70 ± 0.50	3.84 ± 0.60
A	W [mm]	10.46 ± 2.01	10.86 ± 1.23	11.93 ± 2.05	12.29 ± 2.43	13.50 ± 1.29
P		9.47 ± 0.35	10.40 ± 1.14	10.78 ± 2.17	11.00 ± 2.00	12.33 ± 0.58
A	T [mm]	1.04 ± 0.28 *#	1.28 ± 0.20 *#	1.36 ± 0.38 *#	2.60 ± 0.78 *	3.13 ± 0.25 **#**
P		1.30 ± 0.27 #	1.20 ± 0.25 #	1.89 ± 0.78 #	2.22 ± 0.71	3.00 ± 0.89 #

Region of IVD: A—anterior, P—posterior. Geometric parameters: L—length, W—width, T—thickness. * Significant statistical difference between the value at 30 min hydration time and other measurements (*p* < 0.05). # Significant statistical difference between the value at 40 min hydration time and other measurements (*p* < 0.05).

## Data Availability

The original contributions presented in the study are included in the article, further inquiries can be directed to the corresponding author.
